# Combined Effect of Plant Protein Isolate Content and the Homogenization Processes on the Physical Stability of Oily Extract Emulsions

**DOI:** 10.3390/foods14213717

**Published:** 2025-10-30

**Authors:** Juan A. Damas-Espinoza, Liliana Alamilla-Beltrán, Diana E. Leyva-Daniel, Fidel Villalobos-Castillejos, Humberto Hernández-Sánchez, Antonio R. Jiménez-Aparicio

**Affiliations:** 1 Escuela Nacional de Ciencias Biológicas, Instituto Politécnico Nacional, Unidad Profesional Adolfo López Mateos, Av. Wilfrido Massieu s/n, Gustavo A. Madero, Ciudad de México CP 07738, Mexico; jdamasantonio@gmail.com (J.A.D.-E.); hhernandezs@ipn.mx (H.H.-S.); 2 Departamento de Ingeniería Química, Industrial y de Alimentos (DIQIA), Universidad Iberoamericana, Paseo de la Reforma 880, Lomas de Santa Fe, Ciudad de México CP 01219, Mexico; fidel.villalobos@ibero.mx; 3 Centro de Desarrollo de Productos Bióticos, Instituto Politécnico Nacional (CEPROBI-IPN), San Isidro, Yautepec CP 62739, Morelos, Mexico; aaparici@ipn.mx

**Keywords:** oil/water emulsion, microfluidization, rotor–stator, rheology

## Abstract

The homogenization methods and selection of biomaterials of the continuous phase are critical in the formulation of food emulsions. This study evaluated the stability of emulsions containing an oily extract using soy protein isolate (SPI), pea protein isolate (PPI), and two homogenization techniques: microfluidization (MF) and rotor–stator (RS). Emulsions formulated with SPI and processed by MF exhibited the highest stability, with a Turbiscan Stability Index (TSI) of 0.85, a mean droplet size of 160.1 nm, a polydispersity index of 0.152, a ζ-potential of −29.3 mV, and an apparent viscosity of 8.1 mPa·s. The PPI emulsions processed by MF showed slightly higher TSI (1.6) and droplet size (188.1 nm). All MF emulsions achieved desirability >0.8. The RS systems showed lower stability, with a TSI of 5.7 (SPI) and 7.9 (PPI), and droplet sizes >1700 nm, despite more negative ζ-potentials (−40.2 mV for SPI, −36.7 mV for PPI). All optimized emulsions showed pseudoplastic flow behavior, with a transition to Newtonian flow at higher shear rates. Overall, microfluidization significantly improved emulsion stability and rheological properties.

## 1. Introduction

In the food industry, emulsions are widely employed in the formulation and development of a variety of products such as beverages, soups, mayonnaise, dressings, and sauces [1]. The emulsions are a dispersion of two immiscible or partially immiscible liquids, in which one (the dispersed phase) is spread within the other (the continuous phase). The formation of an emulsified system requires the application of energy, which facilitates the homogenization of the phases and leads to the generation of droplets with a controlled and limited size distribution. However, emulsions are thermodynamically unstable systems, susceptible to phase separation due to physical destabilization phenomena. Therefore, understanding the microstructure and phase behavior of emulsified systems is essential for predicting their shelf life and ensuring product stability [2,3]. Achieving a stable emulsion over time is crucial, as high stability enhances the protection of encapsulated bioactive compounds by minimizing physical and chemical changes within the system [1,4,5]. One of the key steps in emulsion formation is the selection of appropriate biomaterials that comprise the continuous phase, which contribute to the interface by protecting and stabilizing the dispersed phase droplets produced during homogenization. Therefore, these materials must possess effective emulsifying properties. Proteins are commonly employed for this purpose due to their amphipathic nature.

In recent years, there has been an increasing interest in using plant-based proteins as natural emulsifiers, largely due to concerns about the cost and sustainability of animal-derived proteins. Both pea and soy proteins demonstrate comparable emulsifying capabilities, which effectively stabilize oil-in-water emulsions [4,6,7,8,9]. Pea protein provides several advantages, including low allergenicity, excellent digestibility, and high levels of essential amino acids such as lysine, threonine, and tryptophan [10,11]. The advantages of soy protein isolate depend on its physicochemical properties, such as emulsification, gelling, and foaming capacity, along with its high biocompatibility and biodegradability, making it suitable for use in food systems [12,13].

Several authors have investigated the relationship between the use of plant proteins as a component of the continuous phase and the stability of emulsified systems, particularly to encapsulate bioactive compounds and preserving their activity. For instance, Oliveira Júnior and Cunha reported a retention rate of 70–75% for the bioaccessibility of trans-resveratrol when encapsulated using plant proteins. Similarly, Fang et al. [12] demonstrated the preservation of the antibacterial activity of riboflavin tetrabutyrate (RTB) when soy protein isolate was used as the encapsulating agent. Additionally, pea protein isolate enhances the stability of emulsions formulated for encapsulating β-carotene and algal oil, as reported by Sun et al. [14] and Yi et al. [15], respectively.

Homogenization is a critical step in the emulsion formation process. It involves the mechanical fragmentation of one liquid into another, resulting in an apparently homogeneous phase [3,16]. Various techniques are available to create emulsions with high physical stability which delay the time it takes for phase separation to occur. One emerging technology is microfluidization, which subjects multiphase fluids to extremely high pressures, reaching up to 200 MPa, as they flow through microchannels. This process leads to protein denaturation, significantly enhancing emulsion stability. The intense shear forces, high-frequency vibrations, pressure differentials, high-velocity impacts, and cavitation effects experienced during microfluidization all contribute to improving the stability of the emulsion [17,18,19,20,21].

In rotor–stator dispersion systems, emulsions are created through highly turbulent flow, which generates eddies of varying sizes within the equipment’s design. These flow dynamics lead to fluid impacts against the chamber walls due to high acceleration and intense shear forces. When used for encapsulating oils or oil-based extracts, rotor–stator homogenization typically produces particles larger than 2 µm. In contrast, microfluidization can achieve particle sizes smaller than 0.1 µm. Additionally, rotor–stator homogenization tends to incorporate a significant amount of oxygen into the continuous phase, which can negatively affect the stability of oily and bioactive cores [4,22,23,24].

This study aims to evaluate the effect of plant protein-based emulsifiers (pea and soy protein isolates) and two homogenization techniques (rotor–stator and microfluidization) on the physicochemical properties, and rheological behavior of oil-in-water emulsions formulated with oily extract of chilhuacle chili (*Capsicum annuum* L.) as a model bioactive extract.

## 2. Materials and Methods

### 2.1. Materials

The continuous phase was formulated using soy protein isolate (SPI) (90% protein content), pea protein isolate (PPI) (85% protein content) Meli-Natura^®^ (Ciudad de México, Mexico), and maltodextrin 10DE donated by Best Ingredients^®^ (Cdad. Santa Catarina, NL, Mexico). For the preparation of the oily extract, chilhuacle chili Sazón Mart^®^ (Oaxaca, Mexico) and corn oil Mazola^®^ (Ciudad de México,Mexico) were used.

### 2.2. Preparation of Chili Extracts Rich in Bioactive Compounds

An oily extract was obtained from chilhuacle chili. First, dried chilies were first selected and cleaned to remove impurities and stems. The cleaned chili samples were then dehydrated at 60 °C for 6 h using a vacuum oven (OV-12 JEIO TECH, Seoul, Republic of Korea) to reduce moisture content and facilitate particle size reduction. The dried chilies were ground using a food grinder (NBR-0601WM, Nutribullet^®^, Los Angeles, CA, USA) and subsequently sieved to obtain a uniform particle size of ≥700 µm. The extraction process was carried out by mixing chilhuacle powder with corn oil at a 1:2 weight-to-volume (*w*/*v*) ratio in a Thermomix^®^ (TM 31, Vorwerk, Wuppertal, Germany) at 60 °C and 1100 rpm for 10 min. The resulting mixture was filtered through gauze and centrifuged at 3700 rpm for 15 min. The final extract was stored in amber glass bottles to protect it from degradation [25]. This oily extract was used solely as a model system to elucidate the emulsions, so its characterization is not presented.

### 2.3. Emulsion Preparation by Microfluidization

Biomaterials comprising the continuous phase at different concentrations were prepared following the methodology described by Villalobos-Castillejos et al. [20] and Pereyra-Castro et al. [2], with minor modifications. The biomaterials for the continuous phase, consisting of plant protein isolate and maltodextrin 10DE, were dispersed in deionized water to achieve a total solids content of 20% (*w*/*w*) and were allowed to hydrate for 12 h at 8 °C. A coarse emulsion was then prepared using a stainless steel two-blade grinder (Model 59765, Hamilton Beach, Glen Allen, VA, USA) operating at 10,000 rpm. The continuous phase was mixed with the chilhuacle oil extract at a ratio of 1:8 (g extract: g solids). This pre-emulsion was subsequently homogenized using a microfluidizer (M-110Y, Microfluidics, Westwood, MA, USA) at pressures ranging from 68.9 to 96.5 MPa. The equipment was fitted with a Y-type interaction chamber containing 75 μm diameter microchannels, followed by an auxiliary Z-type chamber with a 200 μm diameter flow channel. Each formulation was processed with two passes (cycles) through the system under the specified conditions. An ice bath was employed at the equipment outlet to maintain the emulsion at room temperature (25 °C). A combined D-optimal mixture experimental design was utilized to assess the effects of formulation on emulsions produced via microfluidization (Table 1). The same design was used for each of the proteins (SPI and PPI).

### 2.4. Emulsion Preparation by Rotor–Stator

As a comparative method, emulsions were also prepared using rotor–stator homogenization, one of the most widely used techniques for emulsion formation. Biomaterials were dispersed in deionized water at various concentrations to achieve a total solids content of 20% *w*/*w* (Table 2). The dispersions were allowed to hydrate for 12 h at 8 °C. Homogenization was carried out using a rotor–stator homogenizer (T-18 Basic Ultra-Turrax, IKA, Breisgau, Germany) equipped with a S18N-19G (IKA, Germany) dispersion tool, featuring a stator diameter of 19 mm and a rotor diameter of 12.7 mm. Different speed levels were utilized: N2 (7000 rpm), N3 (11,000 rpm), and N4 (15,500 rpm). The oily extract was added dropwise into the aqueous phase, and homogenization was maintained for 3 min at room temperature (25 °C) [25]. A combined D-optimal mixture experimental design was applied for this method (Table 2). The same design was used for each of the proteins (SPI and PPI).

### 2.5. Emulsion Stability by Turbiscan Stability Index

A Turbiscan stability analyzer (Lab Expert, Formulaction Inc., L’Union, France) was used following the methodology reported by Villalobos-Castillejos et al. [20], with minor modifications. The analysis is based on multiple light scattering phenomena using infrared light (λ = 880 nm) and was performed in a cylindrical glass cell (25.1 mm internal diameter × 72.5 mm height).

The Turbiscan Stability Index (TSI) quantifies destabilization phenomena such as sedimentation, creaming, and precipitation. The associated software, EasySoft Turbiscan Converter (V1.1, Formulaction Inc., France), calculates the TSI value based on the average variation in backscattered light. TSI measurements were taken every 20 min over a period of 4 h. Lower TSI values (closer to zero) indicate greater emulsion stability [26].

### 2.6. Determination of Mean Droplet Size and Polydispersity Index

The mean droplet size (MDS) and polydispersity index (PDI) were determined using a particle size analyzer (Zetasizer Nano ZS, Malvern Instruments, Worcestershire, UK), which operates based on dynamic light scattering (DLS) to measure micelle displacement through Brownian motion. Emulsions were diluted 1:1000 with deionized water and placed in a measurement cell, following the methodology described by López-Hernández et al., [27] with minor modifications.

### 2.7. ζ Potential

The ζ potential was measured using a particle analyzer (Zetasizer Nano ZS, Malvern Instruments, UK). A 1:1000 dilution of the emulsion was placed in an electric field generated by a pair of electrodes positioned on either side of the measurement cell. In this field, the micelles in the emulsion achieved a constant electrophoretic velocity [28,29].

### 2.8. Determination of Apparent Viscosity

The apparent viscosity of the emulsions was assessed using a rheometer (RST CC, Brookfield Engineering Labs Inc., Middleboro, MA, USA) equipped with a concentric cylinder geometry (CT-40). The analysis was conducted over a shear rate range of 100 to 1000 s^−1^ for 120 s at room temperature (25 °C). The apparent viscosity values are expressed as [mPa·s] [2].

### 2.9. Modeling Turbiscan Stability Index Data

The modeling of the TSI changes of the optimized samples over time was carried out by means of a kinetic model of the following equation reported by Azuara et al. [30]:

(1) $$TSI=\frac{m_{1}t}{m_{2}+t}$$

where TSI corresponds to the Turbiscan Stability Index at time t; m_1_ is the maximum instability limit of the system at time t_∞_; and m_2_ is the instability parameter related to the destabilization kinetics or the time at which TSI/TSI_max_ = 0.5 is reached.

### 2.10. Rheological Properties of Optimized Emulsions

The dynamic rheological properties of the optimized emulsions were evaluated using a rheometer (Discovery Hybrid Rheometer HR-3, TA Instruments, Castle, DE, USA) equipped with a 60 mm diameter parallel plate geometry. Measurements were performed at a constant temperature of 25 °C using a Peltier plate with a fixed gap of 1 mm. A shear rate ranging from 0.1 to 100 s^−1^ was applied to assess changes in viscosity, which were expressed as [mPa·s] [27].

Additionally, the rheological properties of the continuous phase were also determined for each system prepared under the optimized conditions. The analysis was carried out using the same conditions as those used for the emulsions.

### 2.11. Multivariate Analysis

The oily extract emulsions were prepared as described in Section 2.3 and Section 2.4; each test was performed in duplicate. The results of each test are presented in the Supplementary Materials (Tables S1–S4). To summarize the findings from the analysis of the emulsions, a Principal Component Analysis (PCA) was conducted. This multivariate analysis technique was used to summarize the variability in the data related to the properties of the formulated emulsions as a function of the protein isolate concentration in the continuous phase and the homogenization conditions. The PCA was based on the correlation matrix and executed using Minitab Statistical Software V22, employing autoscaling (standardization) by variable to ensure that all parameters contributed equally to the analysis.

### 2.12. Statistical Analysis

Emulsion optimization was carried out using Design-Expert software (version 13.0.1, Stat-Ease Inc., Minneapolis, MN, USA). The significance of each model term was evaluated through analysis of variance (ANOVA) for each response variable, using a significance level of *p* < 0.05. Additionally, the lack of fit value and the adjusted coefficient of determination (r^2^ adj ≥ 0.8) were used.

The optimal conditions for each emulsified system were established using the desirability function (D), defined as the weighted geometric mean of n individual desirability functions (d_i_), with values ranging from 0 (undesirable response) to 1 (fully desirable response). Optimization was therefore achieved by identifying the factor levels that maximized overall desirability. The selection of the optimal emulsified systems focused on minimizing MDS, polydispersity index PDI, ζ-potential, and TSI, while ensuring that the apparent viscosity remained within the range of 15–20 mPa·s [31,32].

## 3. Results and Discussion

### 3.1. Mean Droplet Size and Polydispersity Index

**Figure 1:**
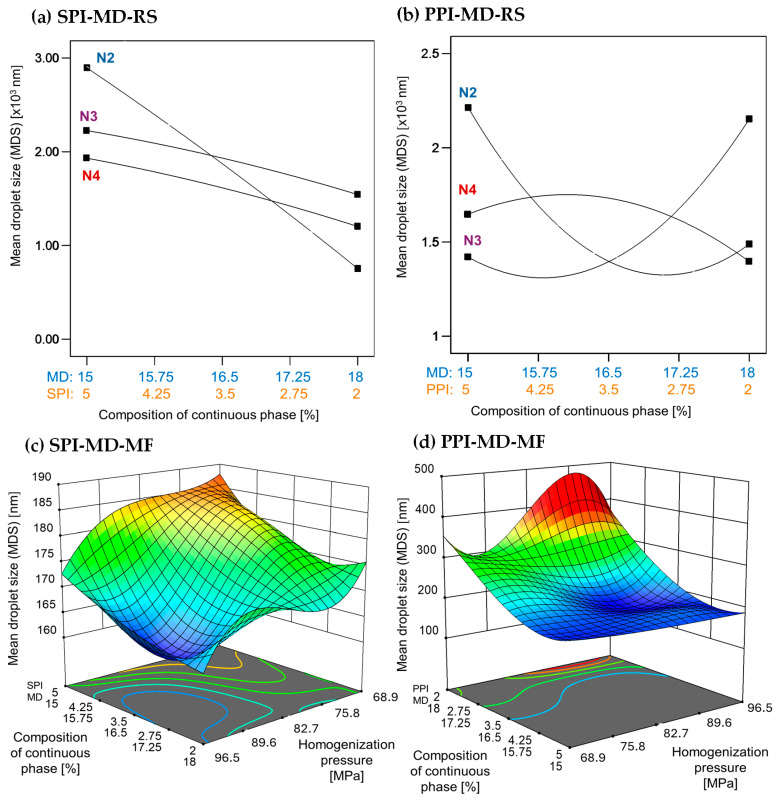
Effect of continuous phase composition and homogenization method on mean droplet size. SPI: soy protein isolate; PPI: pea protein isolate; MD: maltodextrin 10DE; RS: rotor–stator; MF: microfluidization. N2 (7000 rpm), N3 (11,000 rpm), and N4 (15,500 rpm).

The MDS generated during homogenization is crucial for the stability and physicochemical properties of emulsions. Generally, smaller droplet sizes are linked to greater stability [1]. Figure 1 depicts the relationship between droplet size, the concentration and type of protein isolate in the continuous phase, and the homogenization method used.

Emulsions created using rotor–stator homogenization generally showed larger droplet sizes (1000–3000 nm) compared to those produced by microfluidization (100–300 nm), which were approximately ten times smaller (Figure 1). In microfluidized systems, the MDS decreased with increasing homogenization pressure. A high concentration of pea protein isolate (PPI) in the continuous phase led to a further reduction in droplet size. In contrast, soy protein isolate (SPI) demonstrated the opposite effect; droplet size only decreased at lower concentrations (Figure 1c,d).

Previous studies have demonstrated that droplet sizes can vary significantly depending on the formulation and processing conditions employed. Gharsallaoui et al. [33] reported droplet sizes of 1830 ± 0.09 nm in Miglyol 812 emulsions stabilized with pea protein isolate (0.25% *w*/*w*) and 19DE maltodextrin (11% *w*/*w*), homogenized at 50 MPa (500 bar) for three passes. In contrast, Kornet et al. [5] found a smaller average droplet size of 500 ± 0.04 nm in water-in-oil emulsions containing 10% *w*/*w* aqueous phase and stabilized with 2% pea protein concentrate. These emulsions underwent ten homogenization passes at 20 MPa (200 bar).

In the present study, emulsions incorporating SPI achieved significantly smaller droplet sizes ranging from 160 to 190 nm, despite using only two homogenization passes at higher pressure. A similar trend was observed in emulsions stabilized with PPI, where droplet sizes ranged from 180 to 400 nm, with the smallest droplets occurring at a PPI concentration of 5% (*w*/*w*). Homogenization pressure, concentration, and type of protein in the continuous phase are factors that affect interfacial properties, which in turn influence the droplet size of emulsions [34].

**Figure 2:**
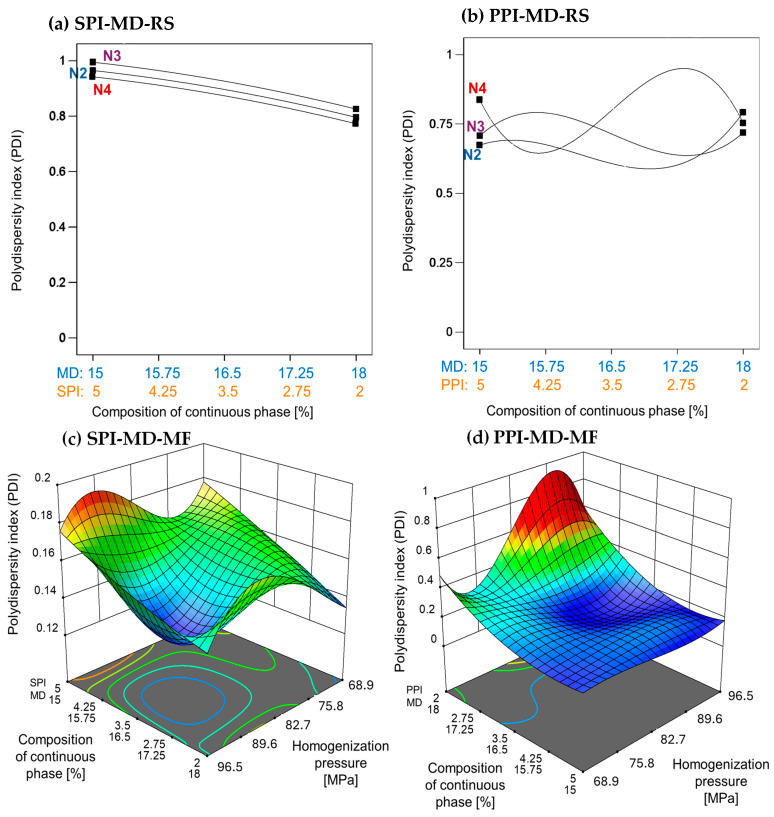
Effect of continuous phase composition and homogenization method on the polydispersity index. SPI: soy protein isolate; PPI: pea protein isolate; MD: maltodextrin 10DE; RS: rotor–stator; MF: microfluidization. N2 (7000 rpm), N3 (11,000 rpm), and N4 (15,500 rpm).

The emulsion processed using the rotor–stator method exhibited a high PDI, with values approaching 1. This indicates a broad droplet size distribution and significant heterogeneity. In the SPI-MD-RS system (Figure 2a), the PDI increased slightly as the SPI concentration rose. In contrast, the PPI-MD-RS system (Figure 2b) exhibited irregular PDI patterns, indicating notable variability in droplet size. These findings underscore the limitations of rotor–stator homogenization for producing stable emulsions under the tested conditions.

Microfluidization significantly reduced the PDI, which was consistent with the observed decrease in MDS. As homogenization pressure increased, the PDI correspondingly decreased (Figure 2c,d), indicating greater uniformity in the droplet size distribution. This inverse relationship demonstrates that higher energy input during microfluidization promotes the formation of more monodisperse emulsion systems.

Villalobos-Castillejos et al. [20] reported notably higher PDI values in rotor–stator-homogenized β-carotene emulsions (ranging from 0.04 to 0.85) compared to microfluidized systems (ranging from 0.191 to 0.612), using maltodextrin (20DE) and gum arabic as stabilizers. This finding supports the superior emulsifying efficiency of microfluidization observed in the present study.

The improvements in MDS and PDI observed in microfluidized emulsions can be attributed to fundamental differences in homogenization mechanisms. While rotor–stator systems primarily depend on shear forces, microfluidization also generates additional effects such as cavitation and turbulence. These combined forces result in more frequent droplet collisions and the formation of interfaces, which are subsequently stabilized by biomaterials in the continuous phase [2,35]. Furthermore, the high-pressure conditions encountered during microfluidization (ranging from 50 to 200 MPa) induce conformational changes in proteins. This exposure of hydrophobic groups enhances interfacial adsorption and stabilization [36].

Maltodextrin plays a crucial role in defining emulsion characteristics. A high content of maltodextrin can lead to flocculation due to depletion interactions, as maltodextrins enhance the attractive forces between droplets. This enhancement can be strong enough to surpass the repulsive forces present. Despite its limited emulsifying capacity, maltodextrin contributes to improved oxidative stability of the encapsulated oil core [33].

### 3.2. Emulsion Stability by Turbiscan Stability Index

**Figure 3:**
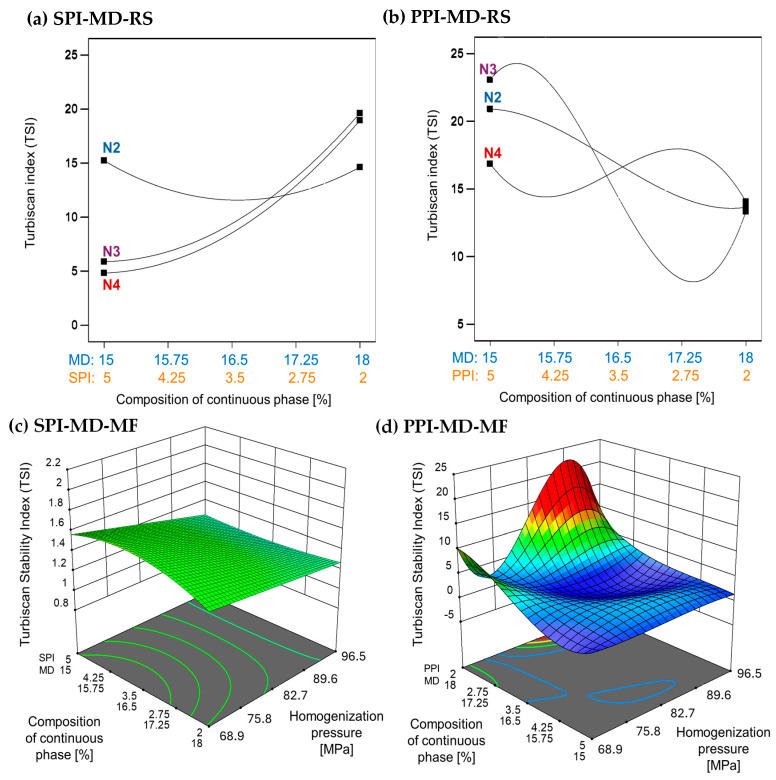
Effect of continuous phase composition and homogenization method on the Turbiscan Stability Index (TSI). SPI: soy protein isolate; PPI: pea protein isolate; MD: maltodextrin 10DE; RS: rotor–stator; MF: microfluidization. N2 (7000 rpm), N3 (11,000 rpm), and N4 (15,500 rpm).

The TSI is a quantitative measure used to evaluate the stability of emulsions. It reflects the system’s resistance to phase separation by analyzing changes in backscattered light over time. Lower TSI values indicate more stable emulsions. Emulsions processed using microfluidization demonstrate improved stability, as indicated by significantly lower TSI values compared to those produced through rotor–stator homogenization [37,38]. In the SPI-MD-RS system, TSI values consistently decreased as the homogenization speed increased above 11,000 rpm (N3). This trend suggests that a higher mechanical energy input results in improved stability (see Figure 3a). In contrast, the PPI-MD-RS system (Figure 3b) exhibited significant instability, and no clear relationship was found between the composition of the continuous phase and the homogenization conditions.

The SPI-MD-MF system (Figure 3c) consistently recorded TSI values below 1.8, demonstrating high emulsion stability. A slight decline in TSI was noted as the homogenization pressure increased. For the PPI-MD-MF system (Figure 3d), increased emulsion stability, indicated by lower TSI values, was observed with higher concentrations of PPI in the continuous phase.

The structural modifications caused by microfluidization reduce viscosity and may also contribute to decreasing the TSI, thereby enhancing emulsion stability. This occurs as the exposure of hydrophobic groups in the protein structure helps stabilize the droplets [39,40]. Phase separation can compromise the structural integrity of the biomaterials in the interface that encapsulate and protect the extract, ultimately diminishing the preservation and effectiveness of the extract’s bioactive components.

According to Stokes’ law, the sedimentation rate of a droplet is proportional to the square of its radius [1]; therefore, the reduction in droplet size and polydispersity observed in oily extract emulsions contributes significantly to enhanced stability. Additional factors, such as increased electrostatic repulsion and higher apparent viscosity, also positively influence the stabilization of these emulsified systems [41].

### 3.3. Electrostatic Stability by ζ Potential

**Figure 4:**
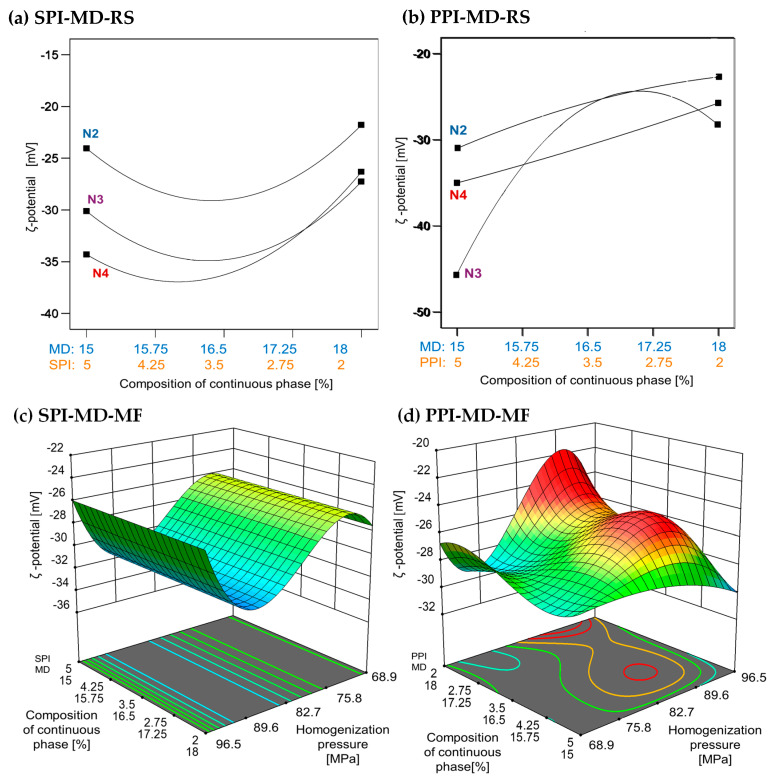
Effect of continuous phase composition and homogenization method on ζ potential. SPI: soy protein isolate; PPI: pea protein isolate; MD: maltodextrin 10DE; RS: rotor–stator; MF: microfluidization. N2 (7000 rpm), N3 (11,000 rpm), and N4 (15,500 rpm).

In oil-in-water emulsion systems, a ζ potential value greater than +30 and less than −30 mV is typically associated with enhanced stability due to strong electrostatic repulsion between droplets formed during homogenization [28]. Higher homogenization speeds and increased concentrations of plant protein isolates (5% *w*/*w*) were associated with lower ζ-potential values. In the SPI-MD-RS system, the lowest ζ potential was observed at a speed of 15,500 rpm with 5% SPI. In contrast, the PPI-based system recorded its minimum ζ potential at the same protein concentration but at a lower speed of 11,000 rpm (see Figure 4a,b). Negative ζ-potential values in oil-in-water emulsions have been previously reported. For example, fish oil emulsions stabilized with soy protein isolate (2% *w*/*w*), both hydrolyzed and non-hydrolyzed, and homogenized via microfluidization, showed ζ-potential values ranging from −52.57 ± 3.26 mV to −48.71 ± 2.40 mV [42]. Similarly, emulsions formulated with sodium caseinate (NaCas) as the encapsulating material exhibited ζ-potential values between −35 and −54 mV [43]. In emulsions prepared with native pea globulins, the ζ-potential ranged from −27.99 to −31.81 mV, with no variations attributed to homogenization pressure [44]. In addition, when a mixture of 20DE maltodextrin and soy protein isolate was used for the encapsulation of chia oil, the stabilized droplets exhibited ζ-potential values between −36.40 and −41.70 mV [2].

The ζ-potential values obtained in this study align with those previously reported, confirming the presence of strong negative surface charges, due to the presence of proteins at the interface, in the emulsions, regardless of the homogenization method. Values below −30 mV are generally associated with increased colloidal stability due to electrostatic repulsion, which minimizes droplet aggregation. The ζ potential reflects the net surface charge and the electrostatic potential at the slipping plane, serving as a key indicator of emulsion stability [45].

### 3.4. Apparent Viscosity

**Figure 5:**
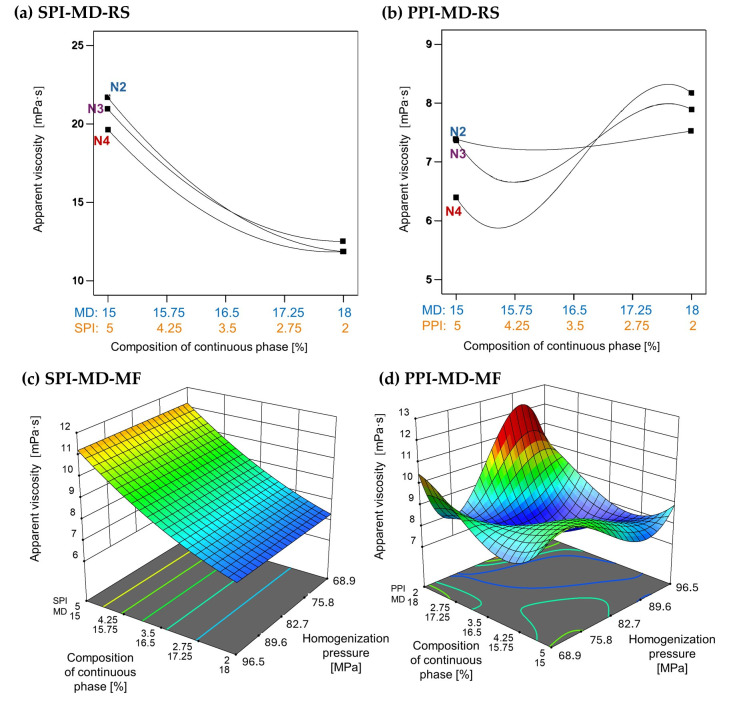
Effect of continuous phase composition and homogenization method on apparent viscosity. SPI: soy protein isolate; PPI: pea protein isolate; MD: maltodextrin 10DE; RS: rotor–stator; MF: microfluidization. N2 (7000 rpm), N3 (11,000 rpm), and N4 (15,500 rpm).

The apparent viscosity of emulsions was strongly influenced by the type and concentration of plant protein isolate used, as well as the homogenization method applied. In emulsions formulated with SPI, as shown in Figure 5a,c, an increase in SPI concentration resulted in higher apparent viscosity. However, microfluidization (SPI-MD-MF, Figure 5c) did not produce an effect on viscosity across the tested conditions. In contrast, emulsions processed via rotor–stator homogenization (SPI-MD-RS, Figure 5a) showed a decrease in viscosity as the homogenization speed increased.

A distinct behavior was noted in emulsions containing PPI. In the PPI-MD-RS system (Figure 5b), the apparent viscosity increased as the concentration of PPI decreased, with the lowest viscosity occurring at the highest PPI concentration (5%) and the maximum homogenization speed (15,500 rpm). Conversely, in microfluidized emulsions (PPI-MD-MF), the viscosity decreased with increasing pressure and higher concentrations of PPI, which is the opposite trend observed in rotor–stator systems.

These differences can be attributed to the protein composition and structural characteristics of the isolates. Kornet et al. [5] state that the high globulin content of PPI promotes aggregate formation, contributing to significant thickening capacity. In contrast, the behavior of SPI during microfluidization aligns with the findings of Pereyra-Castro et al. [2], who reported that high protein concentrations in the continuous phase can enhance intermolecular interactions, leading to structural buildup and increased viscosity.

### 3.5. Model Fitting, Optimization, and Validation

A combined D-optimal mixture experimental design was used to develop predictive models that illustrate how formulation and processing variables, such as homogenization pressure and protein isolate concentration, affect the physicochemical and functional properties of emulsions. This design approach allows for the integration of numerical and categorical factors, which improves the robustness and interpretability of the models (see Table 3 and Table 4). The fit of these models was evaluated using the adjusted coefficient of determination (R^2^). Table 3 and Table 4 show the mathematical models with R^2^ ≥ 0.8. This metric measures the correlation between the observed experimental data and the values predicted by the model. A coefficient of determination close to 1.0 indicates a strong correlation and high predictive accuracy [46]. Reductions in this correlation are linked to system instability, potentially compromising model precision. Nevertheless, the models efficiently identify the effects of independent variables on the dependent responses.

Mathematical models with poor fit (adjusted R^2^ < 0.8) were not determined, as they have limited ability to optimize and predict the behavior of response variables. In Table 5, the actual and predicted values of models from these poorly fitting models are excluded due to their low reliability. In the SPI-MD-RS and PPI-MD-RS systems, the MDS and PDI variables show low predictive accuracy due to various factors, including high system instability, polynomial equation approximations, and a strong correlation between variables. These issues hinder reliable estimation of response values. In contrast, the SPI-MD-MF system demonstrates minimal variation in the TSI under all conditions evaluated. This behavior is like that of the ζ potential, for which no clear relationship between the ζ potential and the composition of the continuous phase is observed. Consequently, there are significant discrepancies between the predicted and actual values in all cases.

Optimization was conducted using a desirability function approach, aiming for values of D greater than 0.8 to maximize the desirability of the model and minimize the response variables. Experiments carried out in triplicate under these optimized conditions confirmed the predictions made by the model. Significant differences (*p* < 0.05) between the observed and predicted values were only found in models where the correlation coefficient was below 0.8 (see Table 5).

**Figure 6:**
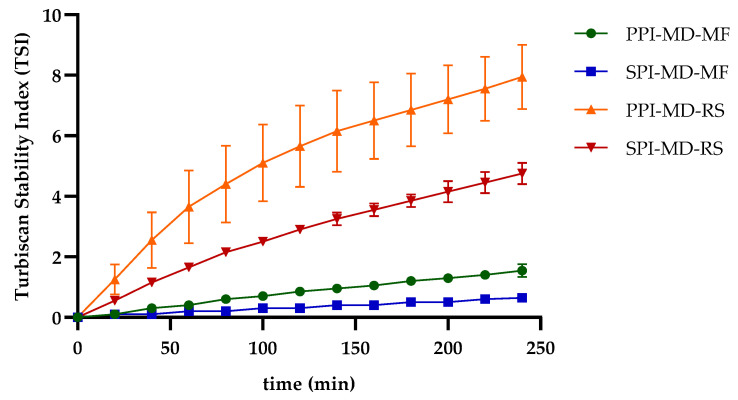
Stability kinetics by Turbiscan Stability Index (TSI) of optimized emulsions.

The kinetics of the TSI (Figure 6) provide insights into the time points at which significant changes in emulsion stability occur. In rotor–stator-homogenized systems, marked variations were observed during the first hour of analysis. In contrast, emulsions processed with microfluidization exhibited enhanced stability, as evidenced by significantly lower TSI values and a more gradual slope over time, indicating reduced rates of destabilization. The pronounced instability typically seen in oil-in-water (O/W) emulsions can be attributed to several factors, including Brownian motion of the dispersed droplets, droplet growth and size heterogeneity, the viscosities of both the continuous and dispersed phases, and electrostatic interactions between the droplets and the surrounding matrix. Moreover, the physicochemical properties and concentration of the encapsulating agents are crucial for emulsion stability. The composition of the dispersed phase also influences stability outcomes; for instance, certain bioactive compounds, such as polyphenols, may compromise emulsion stability by competing with proteins or polysaccharides for interfacial adsorption sites [20,37,38,44,46,47]. The differential distribution of components influences the interaction of each one with the encapsulating matrix, which in turn impacts both the stability and release profile of the active components [48].

### 3.6. Turbiscan Stability Index Modeling of Emulsion Stability Kinetics

The stability kinetics of the optimized emulsions were modeled using a two-parameter kinetic equation. This equation describes the relationship between time and the increase in TSI, which indicates the loss of physical stability over time (Figure 6). This approach also allows for the estimation of the system’s theoretical instability at infinite time (t∞). All models showed excellent goodness of fit, with coefficients of determination (R^2^) exceeding 0.99 (Table 6), confirming that the kinetic model is suitable for describing emulsion destabilization behavior.

The instability constant (m_1_) was found to be lower in emulsions homogenized through microfluidization compared to those processed using rotor–stator homogenization. This indicates that the microfluidized emulsions exhibit more stable behavior, characterized by a slower rate of destabilization. The parameter m_2_, which represents the time needed to reach 50% of the maximum TSI value (TSI/TSI_max_ = 0.5), was observed to increase in the more stable systems. A higher m_2_ value suggests that more time is needed to reach the midpoint of instability, reflecting a greater resistance to destabilization. A similar trend was previously noted by Villalobos-Espinosa et al. [49] in their study of spray-dried emulsions formulated with corn oil and gum arabic as a component of the continuous phase.

### 3.7. Viscosity Behavior of Optimized Emulsions

**Figure 7:**
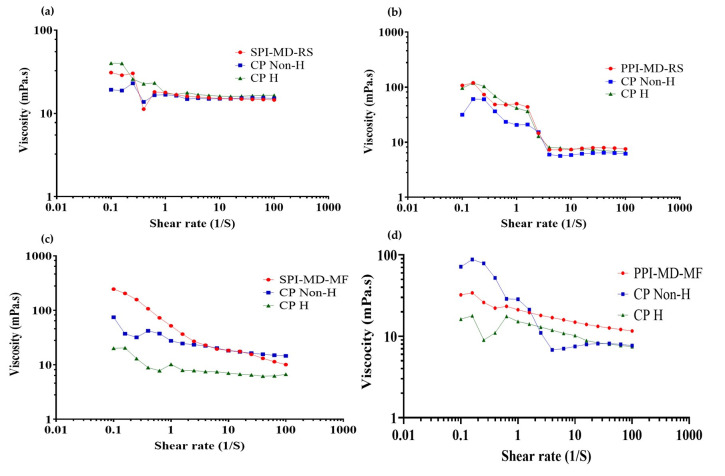
Viscosity vs. shear rate curves of optimized emulsions, non-homogenized and homogenized continuous phase. (**a**) SPI-MD-RS; (**b**) PPI-MD-RS; (**c**) SPI-MD-MF; (**d**) PPI-MD-MF. CP Non-H: non homogenized continuous phase; CP H: homogenized continuous phase.

During homogenization, modifications occur in the physical structure and techno-functional properties of proteins, altering the interactions between components of the continuous and dispersed phases. These changes directly influence the rheological properties of the system [19,50]. To evaluate this effect, flow curves (viscosity vs. shear rate) were obtained for both the optimized emulsions and the corresponding composition of continuous phase formulations under optimized conditions, with and without homogenization treatment (Figure 7).

All emulsified systems exhibited non-Newtonian, pseudoplastic flow behavior. As the shear rate increased, the viscosity decreased. This reduction is attributed to the disruption of the internal structures formed during the homogenization process. The resulting breakdown of these structures leads to shear thinning, which is a characteristic feature of pseudoplastic fluids. At higher shear rates (approximately 1–10 s^−1^), the viscosity of the emulsions tended to stabilize, reaching a nearly constant value. This stabilization indicates a transition toward Newtonian-like behavior under steady-state flow conditions [36,51,52]. The transition to Newtonian-like behavior at high shear rates is due to the possible formation and subsequent breakdown of microaggregates created between the components of the emulsified system (plant protein isolate, maltodextrin 10DE and oily extract). When high shear stress is applied, as in parallel plate rheometry, these microaggregates are gradually disintegrated; this phenomenon has already been reported by several authors. This breakdown reduces the internal resistance to flow, resulting in a constant viscosity profile that is characteristic of Newtonian fluids [53,54].

Emulsions processed with the rotor–stator system (Figure 7a,b) demonstrated greater variability in viscosity and more significant changes in slope compared to those treated with microfluidization (Figure 7c,d). The microfluidized emulsions exhibited smaller variations in viscosity and no sudden changes in slope. This consistent behavior indicates improved dispersion and enhanced stabilization of the system, leading to better flow properties.

In emulsions where PPI was used in the continuous phase, the rheological behavior closely resembled that of the biomaterials in the continuous phase after homogenization. This suggests that homogenization caused structural modifications in the biomaterials that primarily influenced the emulsion’s flow behavior. In contrast, SPI emulsions exhibited viscosity trends more like those of the non-homogenized continuous phase. This indicates that the presence of the oily extract may interfere with or limit the structural reorganization typically induced by homogenization.

### 3.8. Results of Multivariate Analysis

**Figure 8:**
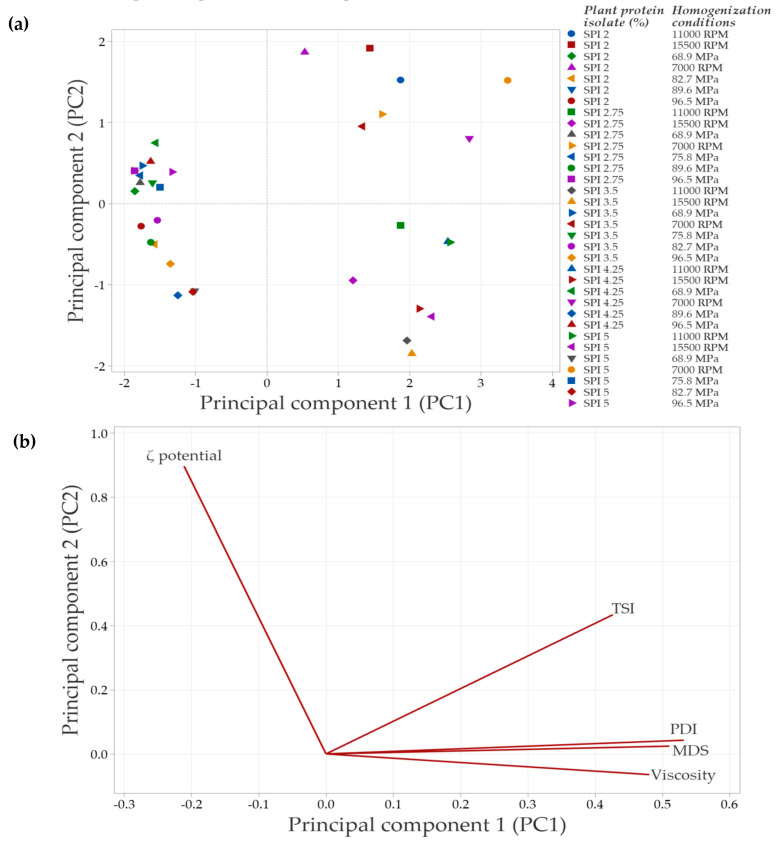
Principal component analysis (PCA) of emulsions prepared with soy protein isolate (SPI) at different concentrations and homogenization conditions. (**a**) Distribution of emulsions based on the concentration of plant protein isolate and the specific homogenization conditions used. (**b**) Correlation of the evaluated response variables with the principal components, shown in a bioplot. Response variables include mean droplet size (MDS), polydispersity index (PDI), Turbiscan Stability Index (TSI), ζ-potential, and apparent viscosity.

In systems with SPI in the continuous phase, the principal component analysis (PCA) (Figure 8) showed that the first two components explained 88.3% of the total variability in the data. Principal component 1 (PC1) accounted for 68.6% of the variability, while principal component 2 (PC2) explained 19.7%.

Figure 8a illustrates the distribution of emulsions under the evaluated conditions. Emulsions clustered in the negative PC1 quadrant correspond to formulations with lower SPI concentrations and less severe homogenization conditions, which have a reduced impact on protein structure. As a result, interfacial interactions are less affected, leading to minor changes in the overall emulsion properties. In contrast, emulsions positioned in the right quadrants (positive PC1 values) arise from treatments that induce significant structural modifications, which are associated with improvements in the evaluated emulsion parameters.

The PCA biplot (Figure 8b) revelated strong correlations among MDS, PDI, TSI, and viscosity, suggesting that an increase in one of these variables is accompanied by corresponding increases in the others. In contrast, the ζ-potential vector points in the opposite direction, indicating an inverse relationship with the variables. Specifically, higher absolute ζ-potential values are associated with improved emulsion stability, as evidenced by decreases in TSI, MDS, and viscosity.

**Figure 9:**
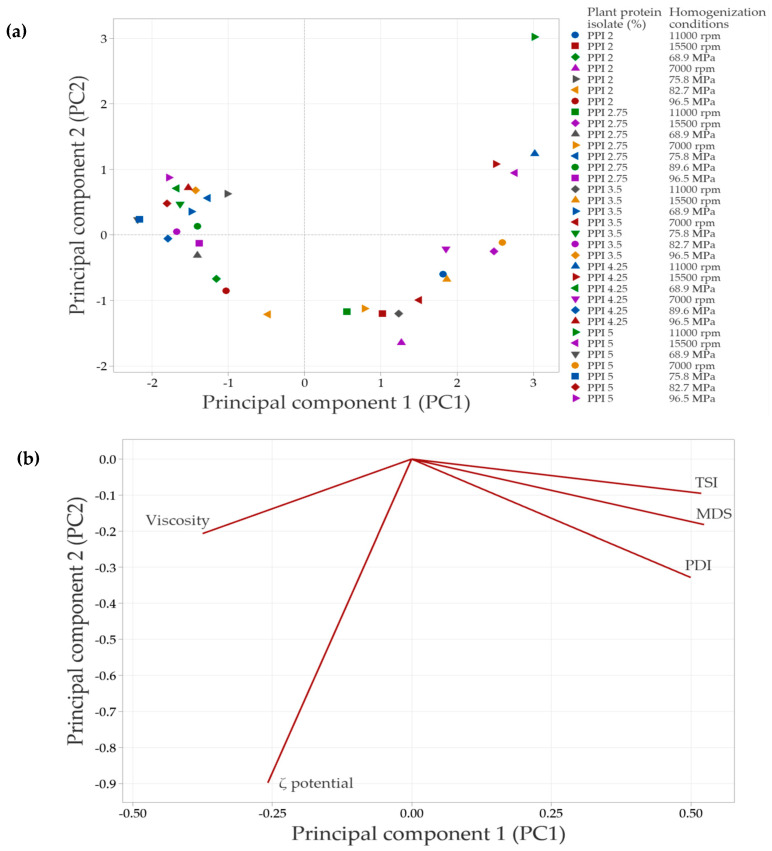
Principal component analysis (PCA) of emulsions prepared with pea protein isolate (PPI) at different concentrations and homogenization conditions. (**a**) Distribution of emulsions based on the concentration of plant protein isolate and the conditions of homogenization. (**b**) Correlation of response variables evaluated with the principal components (bioplot). The response variables include mean droplet size (MDS), polydispersity index (PDI), Turbiscan Stability Index (TSI), ζ-potential and apparent viscosity.

In systems with PPI in the continuous phase, the principal component analysis (PCA) showed that the first two components explained 83.1% of the total variability in the data. Specifically, principal component 1 (PC1) accounted for 65.3% of the variability, while principal component 2 (PC2) explained 17.8%. Similarly to SPI-based systems, emulsions in the negative PC1 quadrant (Figure 8a and Figure 9a) corresponded to lower PPI concentrations and less intense homogenization conditions. A strong correlation was observed among TSI, PDI, and MDS, as indicated by the vectors being closely aligned in the same direction. This suggests that increases in PDI and MDS are associated with an increase in TSI, which leads to decreased emulsion stability. In contrast, viscosity did not align with these variables, indicating a weaker or independent relationship. This result suggests that the viscosity of PPI-based systems in the continuous phase is influenced not only by MDS or PDI but also by additional factors such as protein–protein interactions or solubility differences.

The strong correlation among the test variables identified by the multivariate analysis can be attributed to several factors. Generally, a decrease in the MDS is linked to a reduction in the PDI value of the system, which indicates a more uniform distribution of particle sizes. This behavior is directly related to the physical stability of emulsified systems; when there is a decrease in micelle sizes, there is a decrease in TSI values, which translates into an increase in physical stability, as mentioned above.

This improved stability occurs because the likelihood of instability phenomena, that cause phase separation (such as flocculation, coalescence, and Ostwald ripening), is reduced as the MDS value decreases [55]. The observed reductions in MDS, PDI, and TSI values are a result of the energy applied during the homogenization process. Specifically, microfluidization introduces more energy than rotor–stator homogenization, leading to lower values for these parameters.

The apparent viscosity of emulsified systems is highly dependent on various test variables and the homogenization process used to create the emulsion. Systems emulsified with a rotor–stator using soy protein isolate (SPI) in the continuous phase demonstrate a higher apparent viscosity compared to microfluidized systems. In systems that exhibit coalescence and flocculation phenomena (increase in MDS, PDI, and TSI), there is an increase in viscosity. This increase in viscosity is attributed to the retention of the continuous phase, leading to an effective volume fraction greater than that of the volume fraction of the individual droplets. Conversely, applying a more intense homogenization process, such as microfluidization, minimizes the occurrence of these instability phenomena and results in a decrease in viscosity [1,24].

Additionally, apparent viscosity is also influenced by the interfacial characteristics of the biomaterials present in the continuous phase at the interface. PPI has greater surface hydrophobicity compared to SPI, which increases its capacity to adsorb at the oil-water interface. This increased adsorption at the interface improves the physical stability of the system and helps delay coalescence, ultimately leading to an increase in apparent viscosity [1,56].

The ζ potential has an inverse relationship with the other variables, but at the same time significantly influences the physical stability of emulsions. An increase in the absolute value of the ζ potential is associated with a decrease in the TSI, MDS, and PDI values; these conditions contribute to delaying instability phenomena, since a higher absolute value of the ζ potential favors repulsion between stabilized droplets, thus increasing the physical stability of emulsions [57].

PCA provided a comprehensive view of the interactions among response variables and confirmed the findings obtained through univariate analyses. The strong correlations identified between droplet size distribution, ζ-potential, and stability indices underscore the importance of optimizing both formulation (type and concentration of protein) and processing (homogenization intensity) to design stable emulsions for the encapsulation of bioactive compounds.

## 4. Conclusions

This study demonstrated that microfluidization significantly improved the stability and structural characteristics of emulsions formulated with plant protein isolates and oily extract of chilhuacle chili. Compared with the conventional rotor–stator method, microfluidization produced smaller and more uniform droplets, reduced polydispersity, and lower Turbiscan Stability Index values, all of which are indicators of enhanced physical stability. Soy protein isolate (SPI) conferred superior physical stability due to its well-established emulsifying properties; however, under high-energy homogenization, pea protein isolate (PPI) achieved comparable performance. This highlights the potential of PPI as a sustainable alternative protein emulsifier when combined with optimized processing conditions. The rheological analysis confirmed pseudoplastic flow behavior across all emulsions, with microfluidized systems showing more consistent structural breakdown and uniformity. Importantly, multivariate analysis (PCA) provided an integrated view of the system, revealing strong correlations among droplet size, polydispersity, stability index, and viscosity, while ζ-potential exhibited an inverse relationship with these parameters. These results underscore the interdependence of physicochemical variables and validate microfluidization as a tool to reduce instability phenomena such as coalescence and flocculation. Overall, the findings establish microfluidization as a powerful strategy to enhance emulsion physical stability and functionality, enabling the protection and delivery of lipophilic bioactive compounds. By demonstrating that both SPI and PPI can serve as effective stabilizers under optimized conditions, this work contributes to the development of plant-protein-based systems for functional foods, aligning technological performance with sustainability goals.

## Figures and Tables

**Table 1:** Combined D-optimal mixture experimental design for microfluidization homogenization method.

Experiment	Plant Protein Isolate ^1^ (%)	Maltodextrin (10DE) (%)	Homogenization Pressure (MPa)
1	4.3	15.8	68.9
2	2.8	17.3	68.9
3	5.0	15.0	68.9
4	5.0	15.0	68.9
5	2.0	18.0	68.9
6	3.5	16.5	68.9
7	2.0	18.0	68.9
8	5.0	15.0	75.8
9	3.5	16.5	75.8
10	2.8	17.3	75.8
11	2.0	18.0	75.8
12	5.0	15.0	82.7
13	5.0	15.0	82.7
14	2.0	18.0	82.7
15	3.5	16.5	82.7
16	2.0	18.0	82.7
17	2.8	17.3	89.6
18	4.3	15.8	89.6
19	2.0	18.0	96.5
20	5.0	15.0	96.5
21	3.5	16.5	96.5
22	2.8	17.3	96.5
23	2.0	18.0	96.5
24	4.3	15.8	96.5
25	5.0	15.0	96.5

^1^ Plant protein isolate: soy protein isolate (SPI); pea protein isolate (PPI).

**Table 2:** Combined D-optimal mixture experimental design for rotor–stator homogenization method.

Experiment	Plant Protein Isolate ^1^ (%)	Maltodextrin (10DE) (%)	Homogenization Speed ^2^ (N) (rpm)
1	5.00	15.00	N2
2	5.00	15.00	N2
3	4.25	15.75	N2
4	3.50	16.50	N2
5	2.75	17.25	N2
6	2.00	18.00	N2
7	5.00	15.00	N3
8	5.00	15.00	N3
9	4.25	15.75	N3
10	3.50	16.50	N3
11	2.75	17.25	N3
12	2.00	18.00	N3
13	2.00	18.00	N3
14	5.00	15.00	N4
15	5.00	15.00	N4
16	4.25	15.75	N4
17	3.50	16.50	N4
18	2.75	17.25	N4
19	2.00	18.00	N4
20	2.00	18.00	N4

^1^ Plant protein isolate: soy protein isolate (SPI); pea protein isolate (PPI). ^2^ N2 (7000 rpm), N3 (11,000 rpm), and N4 (15,500 rpm).

**Table 3:** Mathematical models for the SPI-MD-RS and PPI-MD-RS systems obtained by the combined D-optimal mixture design of experiments.

System		Mathematical Models	Adjusted R^2^
SPI-MD-RS	ζ potential	N2: 0.29A + 33.99B − 2.65AB N3: 0.05A + 33.55B − 2.65AB N4: 0.28A + 32.04B − 2.65AB	0.83
	MDS	ND	
	PDI	ND	
	Apparent viscosity	N2: 0.42 + 0.81A + 16.40B − 0.98AB N3: 0.27 + 0.81A + 16.4B − 0.98AB N4: −0.69 + 0.81A + 16.40B − 0.98AB	0.85
	TSI	N2: −3.32A + 542.02B − 47.19AB + 1.19AB(A − B) N3: 6.53A − 500.44B + 43.83AB − 1.17AB(A − B) N4: 2.50A − 6.47B + 0.59AB − 0.06AB(A − B)	0.92
PPI-MD-RS	ζ potential	N2: −1.12A + −10.63B + 0.52AB N3: −3.25A − 71.89B + 4.83AB N4: −0.91A − 2.31B − 0.13AB	0.89
	MDS	ND	
	PDI	ND	
	Apparent viscosity	N2: 0.37A + 8.89B − 0.73AB + 0.02AB(A − B) N3: −0.09A + 81.48B − 7.07AB + 0.18AB(A − B) N4: −0.29A + 108.89B − 9.51AB + 0.24AB(A − B)	0.85
	TSI	N2: 1.62A − 132.67B + 11.9AB − 0.31AB(A − B) N3: 7.56A − 785.48B + 69.38AB − 1.82AB(A − B) N4: −2.99A + 426.846B − 37.31AB + 0.97AB(A − B)	0.87

A: plant protein isolate; B: maltodextrin 10DE; N2 (7000 rpm), N3 (11,000 rpm), and N4 (15,500 rpm). ND: undetermined R^2^ < 0.8.

**Table 4:** Mathematical models for the SPI-MD-MF and PPI-MD-MF systems obtained by the combined D-optimal mixture design of experiments.

System		Mathematical Models	Adjusted R^2^
SPI-MD-MF	ζ potential	ND	
	MDS	35,705.2A + 1527.5B − 2669.3AB + 0.68ABC + 0.001AC^2^ + 1.61 × 10^−9^ABC^3^	0.92
	PDI	81.73A + 2.88B − 5.92AB + 0.002ABC + 6.64 × 10^−8^BC^2^ − 1.34 × 10^−7^ABC^2^ + 3.83 × 10^−12^ABC^3^	0.87
	Apparent viscosity	4.09A + 0.33B − 0.19AB	0.95
	TSI	ND	
PPI-MD-MF	ζ potential	−46,373.1A + 582.02B − 0.16BC − 0.001AC^2^ + 1.4 × 10^−5^BC^2^ + 106.1AB(A − B) − 4.08427 × 10^−10^BC^3^ − 0.03ABC(A − B) + 2.7 × 10^−6^ABC^2^(A − B) − 8.1 × 10^−11^ABC^3^(A − B)	0.93
	MDS	−2.73 × 10^6^A + 34,831.4B + 2.4 × 10^5^AB − 9.06BC − 62.95ABC + 0.001BC^2^ + 0.005ABC^2^ − 2.21 × 10^−8^BC^3^ − 1.72ABC(A − B) − 1.54 × 10^−7^ABC^3^ − 4.21 × 10^−9^ABC^3^(A − B)	0.97
	PDI	−7373.49A + 97.51B + 650.06AB − 0.03BC − 0.17ABC + 2.18 × 10^−6^BC^2^ + 17.81AB(A − B) + 1.48 × 10^−5^ABC^2^ − 6.2 × 10^−11^BC^3^ − 0.005ABC(A − B) − 4.22 × 10^−10^ABC^3^ + 4.04 × 10^−7^ABC^2^(A − B) − 1.15 × 10^−11^ABC^3^(A − B)	0.94
	Apparent viscosity	−38,840.4A + 635.018B + 3428.35AB − 0.17BC − 0.95ABC + 97.04AB(A − B) − 4.22 × 10^−10^BC^3^ − 2.54 × 10^−9^ABC^3^ + 2.39 × 10^−6^ABC^2^(A − B)	0.81
	TSI	−92,394.9A + 2121.1B + 8260.52AB − 0.56BC − 2.31ABC + 4.93 × 10^−5^BC^2^ + 252.29AB(A − B) + 0.001ABC^2^ − 1.42 × 10^−9^BC^3^ − 0.07ABC(A − B) − 6.3 × 10^−9^ABC^3^ + 6.3 × 10^−6^ABC^2^(A − B)	0.97

A: plant protein isolate; B: maltodextrin 10DE; C: homogenization pressure. ND: undetermined R^2^ < 0.8.

**Table 5:** Optimization conditions and validation of the models obtained for the emulsified systems.

System	Conditions (%) ^1^	D		ζ Potential (mV)	MDS (nm)	PDI	Apparent Viscosity (mPa·s)	TSI
SPI-MD-RS	4.0–16.0–N4	0.93	PV	−36.92	ND	ND	15.3	6.4
			AV	−40.2 ± 0.4	ND	ND	14.3 ± 0.2	5.7 ± 0.35
PPI-MD-RS	4.25–15.75–N4	0.61	PV	−36.91	ND	ND	15.0	6.7
			AV	−36.7 ± 1.6	ND	ND	7.7 ± 0.07	7.9 ± 1.06
SPI-MD-MF	3.4–16.4–89.6 MPa	0.87	PV	ND	161.9	0.129	9.0	ND
			AV	ND	160.1 ± 4.2	0.152 ± 0.003	8.1 ± 0.35	ND
PPI-MD-MF	5.0–15.0–96.5 MPa	0.97	PV	−30.0	187.7	0.183	8.9	1.4
			AV	−25.8 ± 0.2	188.1 ± 4.0	0.184 ± 0.005	9.9 ± 0.07	1.6 ± 0.21

D: desirability function; MDS: mean droplet size; PDI: polydispersity index; TSI: Turbiscan Stability Index; PV: predicted value; AV: actual value; ND: undetermined; N4: 15,500 rpm. ^1^ Conditions are expressed as % plant protein isolate—% maltodextrin 10DE—homogenization conditions. SPI: soy protein isolate; PPI: pea protein isolate; MD: maltodextrin 10DE; RS: rotor–stator; MF: microfluidization.

**Table 6:** Results of the modeling adjustment of the stability kinetics.

System	Model Parameters		
	m_1_ (Dimensionless)	m_2_ (h)	R^2^
APCMD-MF	10.41	23.20	0.999
APSMD-MF	10.70	63.29	0.995
APCMD-RS	13.14	2.69	0.999
APSMD-RS	12.50	6.64	0.999

## Data Availability

The original contributions presented in this study are included in the article/Supplementary Materials. Further inquiries can be directed to the corresponding authors.

## Supplementary Materials

The following supporting information can be downloaded at: https://www.mdpi.com/article/10.3390/foods14213717/s1, Table S1. Results of the D-optimal design of mixtures of the system containing soy protein isolate (SPI) in the continuous phase and homogenized by microfluidization; Table S2. Results of the D-optimal design of mixtures of the system containing soy protein isolate (SPI) in the continuous phase and homogenized by rotor–stator; Table S3. Results of the D-optimal design of mixtures of the system containing pea protein isolate (PPI) in the continuous phase and homogenized by microfluidization; Table S4. Results of the D-optimal design of mixtures of the system containing pea protein isolate (PPI) in the continuous phase and homogenized by rotor–stator.

## Abbreviations

The following abbreviations are used in this manuscript:

SPI	soy protein isolate
PPI	pea protein isolate
MD	maltodextrin
TSI	Turbiscan index
MDS	mean droplet size
PDI	polydispersity index
RE	rotor/stator
MF	microfluidization
