# Supplementary material for: Combined Effect of Plant Protein Isolate Content and the Homogenization Processes on the Physical Stability of Oily Extract Emulsions
Source: Foods. 2025 Oct 30;14(21):3717. doi: 10.3390/foods14213717 (PMC12607398; doi:10.3390/foods14213717)
Supplement: Supplementary file 1 [file foods-14-03717-s001.zip › Table S1.docx]

Table S1. Results of the D-optimal design of mixtures of the system containing soy protein isolate (SPI) in the continuous phase and homogenized by microfluidization.

| SPI (%) | MD (10DE) (%) | Homogenization pressure (MPa) | ζ potential (mV) | MDS (nm) | PDI | Viscosity (mPa s) | TSI |
| --- | --- | --- | --- | --- | --- | --- | --- |
| 4.25 | 15.75 | 68.9 | -24.1 ± 6.1 | 181.0 ± 2.1 | 0.171 ± 0.045 | 10.3 ± 1 | 1.6 ± 0.2 |
| 2.75 | 17.25 | 68.9 | -26.2 ± 2.4 | 175.2 ± 2.4 | 0.147 ± 0.124 | 8.1 ± 0.7 | 1.1 ± 0.4 |
| 5 | 15 | 68.9 | -31.9 ± 1.8 | 187.8 ± 1.5 | 0.177 ± 0.016 | 11.4 ± 0.4 | 1.35 ± 0.5 |
| 2 | 18 | 68.9 | -26.7 ± 5.2 | 175.6 ± 1.2 | 0.135 ± 0.002 | 7.4 ± 0.5 | 1.1 ± 0.1 |
| 3.5 | 16.5 | 68.9 | -25.3 ± 1.9 | 173.1 ± 1.7 | 0.155 ± 0.009 | 8.6 ± 0.6 | 1.2 ± 0.1 |
| 5 | 15 | 75.8 | -26.2 ± 1.7 | 185.2 ± 1.9 | 0.166 ± 0.029 | 10.4 ± 0.1 | 0.8 ± 0.01 |
| 3.5 | 16.5 | 75.8 | -26.1 ± 3.1 | 181.2 ± 1.2 | 0.158 ± 0.008 | 9.5 ± 0.1 | 1.0 ± 0.06 |
| 2.75 | 17.25 | 75.8 | -25.9 ± 2.2 | 174.3 ± 2.6 | 0.156 ± 0.001 | 8.0 ± 0.7 | 1.3 ± 0.08 |
| 2 | 18 | 75.8 | -25.6 ± 1.6 | 169.0 ± 2.3 | 0.149 ± 0.078 | 7.7 ± 0.2 | 1.1 ± 0.0 |
| 5 | 15 | 82.7 | -32.0 ± 1.1 | 184.9 ± 1.6 | 0.176 ± 0.006 | 11.1 ± 0.3 | 1.5 ± 0.1 |
| 2 | 18 | 82.7 | -29.8 ± 0.4 | 170.2 ± 1.4 | 0.169 ± 0.001 | 7.4 ± 0.1 | 1.95 ± 0.1 |
| 3.5 | 16.5 | 82.7 | -28.4 ± 0.8 | 170.7 ± 1.6 | 0.140 ± 0.061 | 8.8 ± 0.1 | 1.9 ± 0.02 |
| 2.75 | 17.25 | 89.6 | -29.5 ± 0.9 | 162.5 ± 1.5 | 0.129 ± 0.001 | 7.9 ± 0.6 | 1.5 ± 0.0 |
| 4.25 | 15.75 | 89.6 | -32.0 ± 1.2 | 167.6 ± 1.2 | 0.151 ± 0.003 | 10.1 ± 0.1 | 0.9 ± 0.04 |
| 2 | 18 | 96.5 | -28.6 ± 1.0 | 161.9 ± 2.0 | 0.149 ± 0.005 | 7.1 ± 0 | 1.2 ± 0.1 |
| 5 | 15 | 96.5 | -25.6 ± 0.7 | 173.2 ± 0.7 | 0.175 ± 0.004 | 11.5 ± 0.7 | 1.7 ± 0.0 |
| 3.5 | 16.5 | 96.5 | -30.8 ± 0.5 | 164.4 ± 0.9 | 0.167 ± 0.013 | 8.8 ± 0.5 | 2.1 ± 0.5 |
| 2.75 | 17.25 | 96.5 | -25.7 ± 0.7 | 168.0 ± 1.0 | 0.138 ± 0.026 | 7.6 ± 0.3 | 1.4 ± 0.3 |
| 4.25 | 15.75 | 96.5 | -25.1 ± 0.4 | 167.8 ± 1.1 | 0.169 ± 0.009 | 9.4 ± 0.2 | 1.5 ± 0.2 |
