# Supplementary material for: Combined Effect of Plant Protein Isolate Content and the Homogenization Processes on the Physical Stability of Oily Extract Emulsions
Source: Foods. 2025 Oct 30;14(21):3717. doi: 10.3390/foods14213717 (PMC12607398; doi:10.3390/foods14213717)
Supplement: Supplementary file 1 [file foods-14-03717-s001.zip › Table S2.docx]

Table S2. Results of the D-optimal design of mixtures of the system containing soy protein isolate (SPI) in the continuous phase and homogenized by rotor-stator.

| SPI (%) | MD (10DE) (%) | Homogenization speed (rpm) | ζ potential (mV) | MDS (nm) | PDI | Viscosity (mPa s) | TSI |
| --- | --- | --- | --- | --- | --- | --- | --- |
| 5 | 15 | 7,000 | -25.7 ± 1.4 | 3172.3 ± 258.8 | 0.954 ± 0.052 | 22.4 ± 1.5 | 15.45 ± 1.2 |
| 4.25 | 15.75 | 7,000 | -27.0 ± 2.7 | 3478.0 ± 165.7 | 0.986 ± 0.275 | 15.9 ± 2.2 | 10.3 ± 1.1 |
| 2.75 | 17.25 | 7,000 | -27.3 ± 1.9 | 980.9 ± 149.63 | 0.999 ± 0.167 | 14.1 ± 1.5 | 14.9 ± 2.4 |
| 3.5 | 16.5 | 7,000 | -27.7 ± 1.5 | 1313.0 ± 91.4 | 0.819 ± 0.067 | 13.6 ± 1.3 | 12.9 ± 1.7 |
| 2 | 18 | 7,000 | -23.1 ± 3.7 | 1021.2 ± 103.9 | 0.691 ± 0.004 | 11.7 ± 0.7 | 12.6 ± 3.7 |
| 5 | 15 | 11,000 | -30.5 ± 0.7 | 2160.7 ± 51.1 | 0.995 ± 0.007 | 21.0 ± 2.0 | 5.4 ± 1.3 |
| 4.25 | 15.75 | 11,000 | -32.4 ± 4.3 | 2103.5 ± 49.4 | 0.989 ± 0.049 | 17.8 ± 0.3 | 9.6 ± 0.9 |
| 2.75 | 17.25 | 11,000 | -32 .0 ± 2.1 | 2238.5 ± 87.4 | 0.881 ± 0.216 | 11.8 ± 1.7 | 11.2 ± 0.3 |
| 2 | 18 | 11,000 | -27.1 ± 1.4 | 1359.5 ± 161.2 | 0.848 ± 0.189 | 13.0 ± 1.5 | 20.5 ± 3.5 |
| 3.5 | 16.5 | 11,000 | -37.2 ± 1.3 | 1922.0 ± 209.7 | 0.849 ± 0.340 | 14.0 ± 0.1 | 8.2 ± 1.7 |
| 2.75 | 17.25 | 15,500 | -35.2 ± 1.2 | 1161.6 ± 157.5 | 0.722 ± 0.000 | 11.3 ± 0.4 | 11.9 ± 1.1 |
| 2 | 18 | 15,500 | -25.6 ± 1.4 | 1334.5 ± 50.2 | 0.799 ± 0.187 | 11.7 ± 0.6 | 19.3 ± 0.8 |
| 5 | 15 | 15,500 | -34.5 ± 2.1 | 2029.5 ± 238.3 | 0.937 ± 0.038 | 18.5 ± 1.9 | 5.1 ± 0.8 |
| 4.25 | 15.75 | 15,500 | -34 ± 1.8 | 1657.5 ± 39.2 | 0.931 ± 0.079 | 19.0 ± 0.9 | 5.1 ± 0.9 |
| 3.5 | 16.5 | 15,500 | -38 ± 0.9 | 1545 ± 164.7 | 0.922 ± 0.167 | 14.4 ± 2.4 | 9.3 ± 1.0 |
