# Supplementary material for: Combined Effect of Plant Protein Isolate Content and the Homogenization Processes on the Physical Stability of Oily Extract Emulsions
Source: Foods. 2025 Oct 30;14(21):3717. doi: 10.3390/foods14213717 (PMC12607398; doi:10.3390/foods14213717)
Supplement: Supplementary file 1 [file foods-14-03717-s001.zip › Table S3.docx]

Table S3. Results of the D-optimal design of mixtures of the system containing pea protein isolate (PPI) in the continuous phase and homogenized by microfluidization.

| PPI (%) | MD (10DE) (%) | Homogenization pressure (MPa) | ζ potential (mV) | MDS (nm) | PDI | Viscosity (mPa s) | TSI |
| --- | --- | --- | --- | --- | --- | --- | --- |
| 4.25 | 15.75 | 68.9 | -28.9 ± 0.1 | 205.3 ± 10.3 | 0.191 ± 0.001 | 8.5 ± 0 | 1.3 ± 0.2 |
| 2.75 | 17.25 | 68.9 | -26.8 ± 0.1 | 301.2 ± 3.7 | 0.341 ± 0.001 | 9.9 ± 0.1 | 8.7 ± 0.2 |
| 5 | 15 | 68.9 | -27.8 ± 0 | 203.2 ± 4.5 | 0.188 ± 0.003 | 9.8 ± 0.1 | 1.5 ± 0.1 |
| 2 | 18 | 68.9 | -26.7 ± 0.3 | 355.2 ± 15.2 | 0.494 ± 0.057 | 10.5 ± 0.9 | 10.2 ± 0.4 |
| 3.5 | 16.5 | 68.9 | -27.0 ± 0.4 | 222.8 ± 9.7 | 0.227 ± 0.004 | 7.9 ± 0.7 | 1.9 ± 0.5 |
| 5 | 15 | 75.8 | -27.7 ± 0.6 | 194.1 ± 2.6 | 0.191 ± 0.006 | 9.7 ± 0.7 | 1.2 ± 0.6 |
| 3.5 | 16.5 | 75.8 | -28.0 ± 0.5 | 223.1 ± 3.4 | 0.215 ± 0.006 | 8.5 ± 0.5 | 2.1 ± 0.6 |
| 2.75 | 17.25 | 75.8 | -28.6 ± 0.5 | 276.2 ± 2.8 | 0.283 ± 0.002 | 8.1 ± 0.4 | 2.4 ± 0.2 |
| 2 | 18 | 75.8 | -29.1 ± 0.4 | 304.0 ± 5.7 | 0.325 ± 0.019 | 7.9 ± 0.3 | 3.2 ± 0.3 |
| 5 | 15 | 82.7 | -27.8 ± 0.1 | 191.4 ± 1.4 | 0.174 ± 0.000 | 8.6 ± 0.1 | 1.7 ± 0.1 |
| 2 | 18 | 82.7 | -24.9 ± 0.1 | 403.0 ± 1.6 | 0.661 ± 0.098 | 10.0 ± 0.1 | 14.1 ± 0.2 |
| 3.5 | 16.5 | 82.7 | -25.3 ± 0.1 | 200.9 ± 2.4 | 0.203 ± 0.034 | 7.9 ± 0.2 | 1.5 ± 0.1 |
| 2.75 | 17.25 | 89.6 | -25.8 ± 0.1 | 229.8 ± 3.6 | 0.237 ± 0.005 | 7.7 ± 0.2 | 2.5 ± 0.1 |
| 4.25 | 15.75 | 89.6 | -24.8 ± 0.0 | 188.4 ± 6.4 | 0.183 ± 0.002 | 8.1 ± 0.0 | 1.6 ± 0.1 |
| 2 | 18 | 96.5 | -26.1 ± 0.3 | 367.9 ± 7.7 | 0.597 ± 0.066 | 10.2 ± 0.1 | 8.3 ± 0.6 |
| 5 | 15 | 96.5 | -30.0 ± 0.5 | 185.8 ± 7.2 | 0.180 ± 0.002 | 8.9 ± 0.0 | 1.5 ± 0.2 |
| 3.5 | 16.5 | 96.5 | -28.2 ± 0.2 | 197.0 ± 5.4 | 0.188 ± 0.002 | 7.8 ± 0.1 | 2.5 ± 0.3 |
| 2.75 | 17.25 | 96.5 | -27.4 ± 0.1 | 301.0 ± 6.3 | 0.415 ± 0.001 | 9.4 ± 0.1 | 4.1 ± 0.2 |
| 4.25 | 15.75 | 96.5 | -28.7 ± 0.2 | 200.1 ± 2.7 | 0.195 ± 0.001 | 8.1 ± 0.2 | 1.9 ± 0.1 |
