# Supplementary material for: Combined Effect of Plant Protein Isolate Content and the Homogenization Processes on the Physical Stability of Oily Extract Emulsions
Source: Foods. 2025 Oct 30;14(21):3717. doi: 10.3390/foods14213717 (PMC12607398; doi:10.3390/foods14213717)
Supplement: Supplementary file 1 [file foods-14-03717-s001.zip › Table S4.docx]

Table S4. Results of the D-optimal design of mixtures of the system containing pea protein isolate (PPI) in the continuous phase and homogenized by rotor-stator.

| PPI (%) | MD (10DE) (%) | Homogenization speed (rpm) | ζ potential (mV) | MDS (nm) | PDI | Viscosity (mPa s) | TSI |
| --- | --- | --- | --- | --- | --- | --- | --- |
| 5 | 15 | 7,000 | -30.7 ± 0 | 2232.3 ± 185.3 | 0.678 ± 0.057 | 7.4 ± 0.2 | 21.0 ± 0.8 |
| 4.25 | 15.75 | 7,000 | -28.9 ± 0.1 | 1578.8 ± 148.7 | 0.637 ± 0.004 | 7.3 ± 0.4 | 19.5 ± 1.2 |
| 2.75 | 17.25 | 7,000 | -23.6 ± 1.3 | 1313.0 ± 57.4 | 0.563 ± 0.003 | 7.5 ± 0.3 | 14.3 ± 1.1 |
| 3.5 | 16.5 | 7,000 | -24.9 ± 0.6 | 1493.8 ± 22.4 | 0.666 ± 0.001 | 7.1 ± 0.1 | 17.0 ± 1.0 |
| 2 | 18 | 7,000 | -22.8 ± 1.7 | 1480.3 ± 52.7 | 0.802 ± 0.078 | 7.5 ± 1.1 | 13.7 ± 0.9 |
| 5 | 15 | 11,000 | -45.0 ± 1.4 | 1353.3 ± 72.5 | 0.712 ± 0.027 | 7.3 ± 0.1 | 22.9 ± 0.4 |
| 4.25 | 15.75 | 11,000 | -36.9 ± 2.1 | 1616.8 ± 134.2 | 0.757 ± 0.024 | 7.0 ± 0.7 | 24.3 ± 0.5 |
| 2.75 | 17.25 | 11,000 | -24.3 ± 1.7 | 1540.3 ± 42.6 | 0.605 ± 0.005 | 8.2 ± 0.6 | 10.2 ± 0.3 |
| 2 | 18 | 11,000 | -28.5 ± 0.8 | 2196.4 ± 193.9 | 0.724 ± 0.009 | 7.9 ± 0.1 | 13.1 ± 0.6 |
| 3.5 | 16.5 | 11,000 | -23.5 ± 0.6 | 1265.0 ± 114.5 | 0.764 ± 0.007 | 6.6 ± 0.0 | 11.6 ± 0.0 |
| 2.75 | 17.25 | 15,500 | -31.8 ± 1.3 | 1888.3 ± 61.9 | 0.963 ± 0.001 | 8.1 ± 0.9 | 17.4 ± 0.2 |
| 2 | 18 | 15,500 | -25.1 ± 0.6 | 1357.5 ± 35.4 | 0.752 ± 0.051 | 8.2 ± 0.2 | 14.1 ± 0.6 |
| 5 | 15 | 15,500 | -34.9 ± 0.3 | 1635.5 ± 25.1 | 0.836 ± 0.028 | 6.4 ± 0.1 | 16.9 ± 1.0 |
| 4.25 | 15.75 | 15,500 | -34.3 ± 1.2 | 1897.0 ± 81.4 | 0.661 ± 0.097 | 6.0 ± 0.2 | 13.9 ± 0.9 |
| 3.5 | 16.5 | 15,500 | -26.9 ± 0.1 | 1417.5 ± 203.7 | 0.778 ± 0.102 | 6.9 ± 0.1 | 17.2 ± 0.8 |
